# The Role of K_ACh_ Channels in Atrial Fibrillation

**DOI:** 10.3390/cells13121014

**Published:** 2024-06-10

**Authors:** Vadim Mitrokhin, Nikola Hadzi-Petrushev, Viktor Kazanski, Stanislav Schileyko, Olga Kamkina, Anastasija Rodina, Alexandra Zolotareva, Valentin Zolotarev, Andre Kamkin, Mitko Mladenov

**Affiliations:** 1 Institute of Physiology, Federal State Autonomous Educational Institution of Higher Education “N.I. Pirogov, Russian National Research Medical University” Ministry of Health, 117997 Moscow, Russia; mitrokhin_vm@rsmu.ru (V.M.); kazanskii_ve@rsmu.ru (V.K.); shileiko_sa@rsmu.ru (S.S.); kamkina_ov@rsmu.ru (O.K.); rodina_as@rsmu.ru (A.R.); zolotareva_ad@rsmu.ru (A.Z.); zolotarev_vi@rsmu.ru (V.Z.); andrey.kamkin@rsmu.ru (A.K.); 2 Institute of Biology, Faculty of Natural Sciences and Mathematics, Ss. Cyril and Methodius University, 1000 Skopje, North Macedonia; nikola@pmf.ukim.mk

**Keywords:** atrial fibrillation, K_ACh_ channels, *I*_KACh_ currents, heart

## Abstract

This manuscript explores the intricate role of acetylcholine-activated inward rectifier potassium (K_ACh_) channels in the pathogenesis of atrial fibrillation (AF), a common cardiac arrhythmia. It delves into the molecular and cellular mechanisms that underpin AF, emphasizing the vital function of K_ACh_ channels in modulating the atrial action potential and facilitating arrhythmogenic conditions. This study underscores the dual nature of K_ACh_ activation and its genetic regulation, revealing that specific variations in potassium channel genes, such as Kir3.4 and K_2P_3.1, significantly influence the electrophysiological remodeling associated with AF. Furthermore, this manuscript identifies the crucial role of the K_ACh_-mediated current, *I*_KACh_, in sustaining arrhythmia through facilitating shorter re-entry circuits and stabilizing the re-entrant circuits, particularly in response to vagal nerve stimulation. Experimental findings from animal models, which could not induce AF in the absence of muscarinic activation, highlight the dependency of AF induction on K_ACh_ channel activity. This is complemented by discussions on therapeutic interventions, where K_ACh_ channel blockers have shown promise in AF management. Additionally, this study discusses the broader implications of K_ACh_ channel behavior, including its ubiquitous presence across different cardiac regions and species, contributing to a comprehensive understanding of AF dynamics. The implications of these findings are profound, suggesting that targeting K_ACh_ channels might offer new therapeutic avenues for AF treatment, particularly in cases resistant to conventional approaches. By integrating genetic, cellular, and pharmacological perspectives, this manuscript offers a holistic view of the potential mechanisms and therapeutic targets in AF, making a significant contribution to the field of cardiac arrhythmia research.

## 1. Introduction

### 1.1. Atrial Fibrillation: Mechanisms and Pathophysiology

Atrial fibrillation (AF) is a common cardiac arrhythmia with serious clinical consequences, leading to emergency department admissions in up to 70% of cases during initial observation periods [1]. Advances in understanding arrhythmogenic processes, particularly molecular dynamics, have resulted in improved therapy outcomes [2]. The development and persistence of AF are attributed to rapid focal activities or re-entrant phenomena, where focal activities act as potential initiators or “drivers” of AF. These actions intensify arrhythmia by destabilizing areas prone to re-entry, which is further exacerbated by conditions such as ischemia, inflammation, or dilatation [2,3]. For focal ectopic activities to effectively initiate rapid “driver” activities and subsequently induce the characteristic fibrillary conduction of AF, they must possess sufficient intensity to provoke irregular activity through areas of heterogeneous conductivity [3].

Re-entrant excitation within the atrial myocardium is closely associated with heterogeneity in the refractory periods among adjacent atrial tissues, contributing to the complexity of AF [4]. Clinically, AF is classified into four distinct forms: paroxysmal, which is self-terminating; persistent, lasting beyond seven days but less than a year, with or without intervention; long-standing persistent, where episodes persist for more than a year; and permanent, where the condition continues despite medical attempts to rectify it. Focal activations are believed to predominantly underlie paroxysmal AF, while re-entrant activities are thought to contribute more significantly to the persistent forms of AF [4].

Additionally, AF is often a consequence of atrial remodeling, which is defined as structural or electrophysiological alteration within the atrial tissues [5]. The concept of atrial remodeling elucidates the pathophysiological foundation of AF, illustrating the intricate mechanisms underlying its diverse clinical forms as well as the problems that come with its management.

### 1.2. Ion Channels and Pathophysiology of AF

Ion channels, particularly potassium (K^+^) channels, are essential for the proper function of excitable tissues, such as the cardiac muscle. They mediate the repolarization of the cardiac action potential (AP), establish the resting membrane potential (RMP), and modulate electrical activity in response to hormonal and metabolic shifts [6]. The acetylcholine (ACh)-activated inward rectifier potassium (K_ACh_) channel is particularly vital for the late phase of repolarization in the context of AF. Genetic variations in the inward rectifier potassium channels (Kir3.4 and K_2P_3.1), both specific atrial channels, along with the dysregulation of cardiac ion channels, including *I*_to_, *I*_Kur_, *I*_Ca,L_, *I*_SK_, *I*_K1_, *I*_KACh_, and *I*_K2P_, are crucial to the electrophysiological remodeling associated with AF [7]. The ACh-induced activation of K_ACh_ channels in atrial myocytes, leading to a reduction in the action potential duration (APD) and hyperpolarization of the RMP, facilitates smaller and stabile re-entrant circuits, perpetuating arrhythmia [8].

The importance of K_ACh_ in AF induction was elucidated in experiments where AF could not be induced through programmed stimulation or burst pacing in the absence of the muscarinic agonist carbachol, in both normal and Kir3.4 knockout (KO) mice [9]. However, following carbachol infusion, AF was successfully induced in control mice, while KO mice remained unaffected, emphasizing K_ACh_’s pivotal role in the vagal nerve-mediated role of AF [9]. Furthermore, mathematical models and experimental observations across various species suggest the inward rectifier K^+^ current, such as that it is mediated by K_ACh_, as a key facilitator of AF [10]. The cholinergic suppression of electrical activity in the atrial myocardium and the inhibitory effect of Ba^2+^ (a K_ACh_ blocker) on this cholinergic reduction of the AP, represent another indicator of the potential role of K_ACh_ in initiating atrial arrhythmias [11].

At the cellular level, AF is associated with pronounced changes, including the shortening and impaired rate of adaptation of APD, modifications in AP morphology, and altered gene expressions of the L-type Ca^2+^ (Ca,_L_) channel and various potassium channels. These changes lead to hyperpolarization of atrial cardiomyocytes and enhanced K_ACh_, significantly affecting voltage-dependent Na^+^ current (*I*_Na_) inactivation. *I*_KACh_ thus plays a more substantial role in sustaining AF compared to other ionic currents, such as those mediated by reduced L-type Ca^2+^ currents [12]. The cholinergic suppression of electrical activity observed in the atrial myocardium across various species and the protective role of K_ACh_ blockers propose *I*_KACh_ activation as a crucial mechanism in the initiation of atrial arrhythmias [11].

### 1.3. Ubiquitous Nature of I_KACh_ and Its Role in Arrhythmogenesis

Emerging research elucidates that the influence of ACh extends beyond the atria to the ventricles, encompassing a variety of species, including humans, cats, and guinea pigs [13]. The experimental administration of ACh to isolated cardiomyocytes induces a directionally rectified current–voltage characteristic, as observed through patch clamp techniques. This finding underscores the widespread role of ACh in cardiac tissues [13].

Studies utilizing animal models further highlight the contributory role of vagus nerve stimulation in the genesis of AF, with cardiac denervation acting to inhibit AF induction. This dualistic effect is also observed in knockout mouse models, where the activation of K_ACh_ channels predisposes them toward AF initiation, while their absence reduces such susceptibility. This underscores the critical role of K_ACh_ in the pathogenesis of AF. The potential therapeutic implications of targeting K_ACh_ channels are significant, particularly with specific K_ACh_ blockers that show promise for AF treatment without adverse effects on the atrioventricular node or ventricles. This suggests a selective distribution of these channels and receptors within the cardiac tissue [9]. The selective distribution of K_ACh_ channels correlates with findings that link AF occurrence to channel activation mediated through M_2_ muscarinic and adrenergic receptors. Muscarinic receptor blockers, such as AF-DX-116, have demonstrated efficacy in preventing AF in canine models. This is contrary to the ineffectiveness of adenosine A1 receptor agonists like 8-cyclopentyl-1,3-dipropylxanthine (DPCPX), further highlighting the specific pathways involved in AF pathophysiology and potential intervention strategies [14].

### 1.4. Channel Density Variations and Cardiac Arrhythmias

In patients without cardiac arrhythmias, the density of the channels responsible for the G protein-coupled inwardly rectifying potassium current (GIRK) was found to be approximately half compared to that in isolated cardiomyocytes from patients afflicted with AF. This observation is accompanied by a concomitant decrease in GIRK4 mRNA levels, a critical subunit of this channel type. These findings suggest an adaptive response in atrial cardiomyocytes to chronic arrhythmogenic conditions by reducing *I*_KACh_ levels, potentially as a compensatory mechanism for alterations in the atrial effective refractory period [8]. Additionally, the differential densities of these channels have been observed between the right and left atria. Research involving the sheep heart has shown that a higher channel density in the left atrium contributes to greater Ach-induced channel acceleration. This finding further elucidates the dose-dependent influence of ACh on the incidence of AF, highlighting the significant role of channel density variations in the pathophysiology of cardiac arrhythmias [15].

### 1.5. Mechanisms Underlying Constitutive K_ACh_ Activity and Its Regulation

Nucleoside diphosphate kinases (NDPKs) are pivotal to the cellular energy transfer process. They are traditionally recognized for their ability to catalyze the transfer of phosphates from adenosine triphosphate (ATP) to guanosine triphosphate (GDP) through a ping-pong mechanism. This enzymatic function is essential for replenishing the GTP pool necessary for sustained G-protein cycling [16]. Notably, it has been observed that atropine, a muscarinic receptor antagonist, does not inhibit the constitutive activity of K_ACh_ in atrial myocytes, suggesting the presence of receptor-independent mechanisms governing constitutive K_ACh_ channel activation [17].

**Figure 1:**
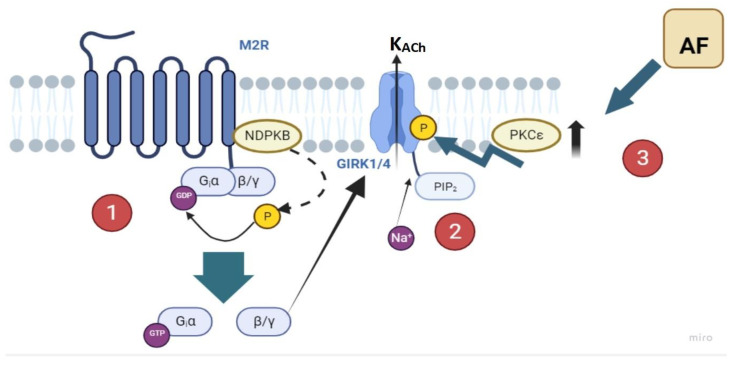
Molecular signaling pathways leading to atrial fibrillation via K_ACh_ channels. The figure illustrates the cascade of events starting with the activation of muscarinic acetylcholine receptors (M_2_R), leading to the activation of acetylcholine-activated inward rectifier potassium channels (K_ACh_). (1) The M_2_R activation prompts G-protein signaling involving GDP-GTP exchange on the Gα subunit and activation of β/γ subunits. (2) This, in turn, stimulates GIRK1/4 channels and leads to the modification of the ion flow, including potassium and sodium. Nucleoside diphosphate kinase B (NDPKB) also plays a role in this pathway. (3) The phosphorylation of the K_ACh_ channels mediated by protein kinase C epsilon (PKCε) is indicated, which contributes to the pathophysiology of atrial fibrillation. This detailed pathway underscores potential therapeutic targets in the treatment and management of AF.

Further investigations have identified that the NDPK B isoform is capable of directly phosphorylating the Gβγ subunit of the G-protein complex, independent of receptor stimulation. This phosphorylation leads to the immediate transfer of phosphate to GDP, which then binds as GTP to the Gα-subunit, thereby activating the G-protein through an indirect, receptor-independent pathway (Figure 1) [18]. Increased expression of NDPK B has been documented in patients with chronic AF, highlighting its critical role in promoting constitutive K_ACh_ activity [19].

Additionally, phosphatidylinositol 4,5-bisphosphate (PIP_2_) has been implicated in the regulation of constitutive K_ACh_ activity (Figure 1). The depletion of PIP_2_ correlates with reduced constitutive *I*_KACh_ currents, indicating that K_ACh_ channels rely on PIP_2_ for maintaining their electrophysiological properties. Elevated PIP_2_ levels within the K_ACh_ channel microdomain have been shown to activate these channels (Figure 1) [20]. Furthermore, sodium ions may enhance the interaction between PIP_2_ and the Kir3.4 subunits of the K_ACh_ channels, suggesting a sodium-mediated modulation of channel activity [21].

There is also evidence of the muscarinic M_1_ and M_3_ Gαq-coupled receptors in the atria [22,23,24]. The activation of these receptors may lead to phospholipase C (PLC)-mediated PIP_2_ depletion, contributing to the rapid desensitization of M_2_-receptor activated *I*_KACh_ currents [25]. Moreover, recent research suggests that internalization of the M_2_R may contribute to the slow desensitization of *I*_KACh_ currents [26], further complexifying the regulatory landscape of atrial electrophysiology.

## 2. Ion Channel Specificity and Regulation in AF

### 2.1. I_KACh_ and K^+^ Channel Heterogeneity in AF: Implications for Tachycardia, Contractile Dysfunction, Autoimmunity, and Therapeutic Intervention

Recent studies have elucidated the differential expression of voltage and time-dependent K^+^ channels across the left and right atria, the implications of which extend to AP repolarization and the RMP [27,28]. Such expression heterogeneity potentially leads to a dispersion in refractory periods, thus facilitating atrial re-entry, a pivotal mechanism in AF development [29]. Investigations, including those conducted by Lomax et al., have delved into the heterogeneity of K_ACh_ expression in adult mouse atria, uncovering a gradient in the *I*_KACh_ current density that suggests a predisposition toward re-entry-induced AF [30]. Additionally, chronic (persistent) AF-associated atrial remodeling has been observed to mediate K_ACh_ downregulation, consequently diminishing muscarinic receptor-mediated atrial AP shortening [17,31]. This downregulation, marked specifically by decreased Kir3.1 and Kir3.4 subunit expressions in AF patients, serves as a potentially protective mechanism against the electrical remodeling-induced shortening of the atrial effective refractory period. AF-promoted atrial remodeling further results in an increase in the constitutively activated ACh-regulated current, showing the complex interplay between AF and atrial electrical alterations [8]. Moreover, *I*_KACh_’s independent activity, albeit with a significantly lower opening frequency in the absence of an agonist, is pronouncedly enhanced in atrial myocytes during AF, contrasting with its minimal activity in healthy hearts [32]. This increase in the constitutive *I*_KACh_ activity, contributing to APD shortening, typifies AF-related electrical remodeling [33]. Canine model studies have substantiated *I*_KACh’_s significant contribution to atrial tachycardia (AT)-related remodeling effects [34]. Research by Dobrev et al. has indicated the prevalence of constitutive *I*_KACh_ openings during chronic AF, a rarity in myocytes from the sinus rhythm (SR) group, elucidating the emergence and persistence of the constitutive *I*_KACh_ activity in chronic AF conditions [17]. This highlights elevated basal current levels exclusively in chronic AF, whereas muscarinic receptor-activated *I*_KACh_ exhibits diminished activity in both paroxysmal AF and chronic AF, emphasizing the sustained nature of *I*_KACh_ activity under chronic AF scenarios [17,31]. AT-induced electrical remodeling constitutes a critical aspect of the pathophysiology underlying AF. In canine models mimicking AF conditions, AT has been shown to precipitate an increase in the agonist-independent constitutive activity of the *I*_KACh_ while concurrently diminishing the current induced by cholinergic stimulation. This observation proposes that the development of constitutive *I*_KACh_ in chronic AF patients may primarily result from a higher atrial rate, regardless of any specific underlying heart disease [35,36]. Moreover, the single-channel characteristics of the constitutive *I*_KACh_ in cardiomyocytes from dogs subjected to AT resemble those identified in chronic AF patients, suggesting a common molecular foundation [37]. Further research into the blockade of the constitutive *I*_KACh_, specifically studies conducted by Bingen and co., elucidates the therapeutic potential of targeting constitutive *I*_KACh_ and the downregulation of Kir3.x channels [38]. This intervention has been found to be effective in terminating re-entry mechanisms by prolonging the APD and altering the restitution slopes of APD and conduction velocity. Such modifications are instrumental in diminishing the probability of APD alternans and channel destabilization, underlining the pivotal role that atrium-specific Kir3.x channels play in modulating fibrillation dynamics [38]. Additionally, the enhanced activity of constitutive *I*_KACh_ is implicated in the contractile dysfunction typical of AF, particularly during AT-induced remodeling. Suppressing constitutive *I*_KACh_ activity significantly improves AT-induced atrial hypo-contractility, attributing the negative consequences for contractile function predominantly to diminished calcium mobilization. This emphasizes the substantial consequences of the constitutive *I*_KACh_ activity not only in the electrophysiological alterations associated with AF but also in the mechanical dysfunction resulting from AT remodeling [37].

Additionally, recent investigations have unveiled a prevalent autoantibody response targeting the Kir3.4 proteins in patients with AF, detectible even prior to the clinical manifestation of the disorder. Utilizing functional analyses of human-induced pluripotent stem cell derived atrial cardiomyocytes, it has been demonstrated that anti-Kir3.4 immunoglobulin G (IgG) isolated from AF patients not only precipitates a reduction in APD but also increases the constitutive *I*_KACh_ [39]. These phenomena are recognized as pivotal contributors to the pathogenesis of AF. Electrophysiological studies in a mouse model engineered to exhibit Kir3.4 autoimmunity have corroborated that Kir3.4 autoantibodies significantly reduce the atrial effective refractory period, consequently elevating the propensity for AF development [21].

### 2.2. Mechanism of Action of K_ACh_ Channels

The activation of K_ACh_ channels is initiated by the stimulation of specific G-protein-coupled receptors, particularly the M_2_ receptors. This stimulation prompts the dissociation of heterotrimeric G_i_ proteins into Gα and Gβγ subunits. Direct binding of the Gβγ subunits to the K_ACh_ channels subsequently triggers their activation [40,41]. Heterotrimeric G-proteins comprise a GDP/GTP-binding Gα subunit and a Gβγ dimer (Figure 1). The activation of G-protein-coupled receptors facilitates the exchange of GDP for GTP on the Gα subunit, leading to the dissociation of Gα from Gβγ and the initiation of various intracellular signaling cascades. The intrinsic GTPase activity of the Gα subunit hydrolyzes GTP back into GDP, permitting the reassociation of the G-protein subunits into their original, inactive state. Gα subunits are classified according to their functional impacts: Gα_s_ stimulates adenylate cyclase, Gα_i_ inhibits adenylate cyclase, and Gα_q_ activates phospholipase C [42]. The Gα_i_ subunit plays a pivotal role in the suppression of adenylyl cyclase, leading to reduced cyclic adenosine monophosphate (cAMP) levels, which indirectly modulate cardiac contractility and are crucial to regulating K_ACh_ channel activity (Figure 1) [43]. In its resting state, the GDP-bound Gα_i_ subunit associates with the Kir3.1 channel subunit, forming a “preformed complex” that enables rapid and efficient activation of the K_ACh_ channels upon Gα_i_ dissociation [44]. This specific interaction highlights the precise linkage between receptor stimulation and subsequent K_ACh_ channel activation. Additionally, the GDP-bound Gα_i_ acts as a Gβγ scavenger, sequestering Gβγ subunits away from K_ACh_ channels and thus preventing agonist-independent channel activity. This mechanism underlines the sophisticated regulatory architecture governing cardiac electrophysiological responses [43].

### 2.3. Modulation of the M_2_-G-Protein K_ACh_ Signaling Pathway

The M_2_-G-protein K_ACh_ signaling pathway predominantly undergoes modulation through phosphorylation, a process significantly mediated by G-protein-coupled receptor kinases (GRKs). GRKs are instrumental in facilitating the uncoupling of M_2_ receptors (M_2_R) from the G-protein, effectively contributing to the attenuation of *I*_KACh_ currents [41]. Additionally, the internalization of M_2_Rs is a critical component of the desensitization of the *I*_KACh_ current, underscoring the significance of receptor endocytosis in the regulation of signaling efficiency [26]. Recent studies have further elucidated that the modulation of *I*_KACh_ can be influenced by mechanisms that are both ligand-binding and voltage-dependent [45,46]. Research by López-Serrano et al. revealed that membrane hyperpolarization significantly enhances the affinity of M_2_R for a super agonist (IXO), consequently increasing the probability of K_ACh_ channel opening. In contrast, depolarization leads to a reduction in the channel’s opening probability, illustrating a dynamic interplay between the membrane potential and receptor–channel interactions [44]. This complex modulation mechanism elucidates the intricate balance and finely tuned control within the cardiac electrophysiological milieu.

### 2.4. Regulation of G_i_-Protein Cycling

The cycling of G_i_-proteins is governed by a series of complex regulatory mechanisms, prominently involving the action of regulators of G-protein signaling proteins (RGS). These proteins are critical to modulating G-protein-coupled receptor (GPCR) effector responses by accelerating the GTPase activity of Gα subunits, thereby influencing both the magnitude and the duration of signaling responses. RGS4 and RGS6 are particularly significant in the cholinergic control of the heart rate. RGS4 is predominantly located in the sinoatrial node (SAN), atrioventricular (AV) region, and atria, displaying GTPase activating protein (GAP) specificity for the Gα_i/o_ and Gα_q/11_ subunits [47,48]. Under conditions of basal vagal tone, RGS4 has a minimal impact on K_ACh_ regulation within the SAN. However, under increased vagal tone, RGS4 plays a crucial role in mitigating excessive *I*_KACh_ currents [49,50]. Phosphatidylinositol-3,4,5-trisphosphate (PIP3) has been shown to bind to RGS4, inhibiting its GAP activity [51]. Conversely, calmodulin, which acts as a Ca^2+^ sensor, can bind directly to RGS4, counteracting the inhibition induced by PIP3 and thereby restoring GAP activity. This interaction facilitates a voltage-dependent control over the G-protein cycle [52,53]. On the other hand, RGS6 acts as a negative regulator of G-protein activation of GIRK channels, which are essential for modulating M_2_R signaling in cardiomyocytes and sinoatrial cells (Figure 1). The RGS6/Gβ5 protein complex serves as a pivotal regulator of M_2_R-K_ACh_ signaling, capable of physically associating with K_ACh_ channels. Genetic deletion of the RGS6 gene in mice has been shown to significantly enhance parasympathetic control of the heart rate, demonstrating its pivotal role in cardiac autonomic regulation [54].

### 2.5. Influence of Na^+^ Concentration on G-Protein Cycling

The concentration of Na^+^ within the physiological range has a evident effect on the G-protein cycling process. Studies have indicated that GIRK channel mutants, which lack the capacity for fast direct Na^+^ regulation, exhibit a slower activation profile (Figure 1). This activation occurs through an indirect pathway associated with Gβγ subunits. Notably, this form of slow channel activation does not necessitate the presence of GTP but is instead inhibited by Gβγ, suggesting a mechanism where Na^+^ ions facilitate the dissociation of GαGDP from Gβγ, thereby enabling the activation of G-protein-gated potassium channels [55,56]. This hypothesis has been supported by quantitative analyses, which have further proven the pivotal role of Na^+^ ions in modulating G-protein signaling pathways [57].

### 2.6. Phosphorylation and PKC Isoforms in Constitutive K_ACh_ Activation

**Figure 2:**
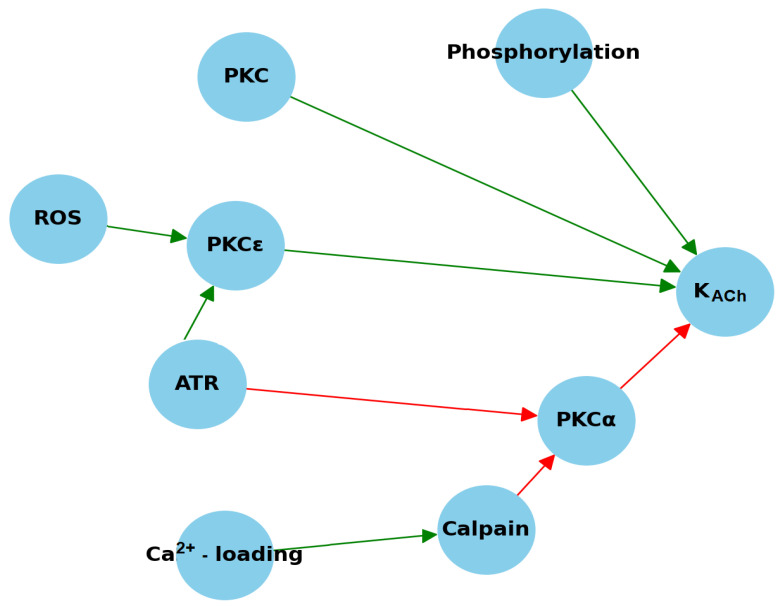
Impact of phosphorylation and PKC isoforms on constitutive K_ACh_ activation. ATR—atrial tachycardia-induced remodeling; PKC—protein kinase C; PKCα—protein kinase C alpha; PKCε—protein kinase C epsilon; ROS—reactive oxygen species. Red arrows represent downregulation, while green arrows indicate upregulation.

Phosphorylation is a critical regulatory mechanism in the development of constitutive K_ACh_ activity (Figure 2). Research conducted by Voigt et al. revealed that the inhibition of protein kinase C (PKC) leads to a significant reduction in the basal current that includes K_ACh_ in patients with chronic AF [31]. Single-channel patch-clamp experiments performed in the inside-out configuration—where the sarcolemma attached to the pipette contained K_ACh_ channels—demonstrated a substantial decrease in the open probability of these channels. This observation suggests that an essential intracellular component crucial for constitutive K_ACh_ activity is lost during this experimental protocol [58].

The expression of PKCε, which is known to stimulate K_ACh_, is significantly increased in patients with chronic AF, while the levels of other PKC isoforms remain unchanged [31,58] (Figure 2). This imbalance underscores the significant role of specific PKC isoforms in the development of constitutive K_ACh_. In dogs with AT-induced remodeling (ATR), a decreased expression of PKCα (conventional PKC isoform) was noted, along with an increased membrane translocation of PKCε (a novel PKC isoform). This differential expression elucidates the distinct roles of PKC isoforms in AF pathology, with a reduction in the tonic inhibitory effect of PKCα and an enhancement of the stimulatory impact of PKCε being key determinants of increased constitutive K_ACh_ activity in AF, Figure 2, [58]. Cellular in vitro pacing experiments have suggested that AT-induced Ca^2+^ loading and subsequent activation of the Ca^2+^-dependent protease calpain may lead to the downregulation of PKCα, potentially increasing the degradation of this isoform [59,60]. Further studies indicate that reactive oxygen species (ROS) not only activate PKCε and promote its translocation to the cell membrane but also that PKCε-dependent constitutive K_ACh_ activity contributes to the perpetuation of AF in the aging heart [61]. These insights highlight the complex interplay between phosphorylation pathways and their modulation by cellular stress responses in the regulation of cardiac electrophysiology.

### 2.7. Impact of miR-30d and External Factors on K_ACh_

**Figure 3:**
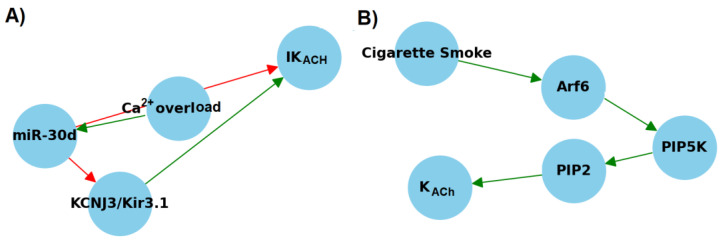
Impact of miR-30d (**A**) and external factors (**B**) on K_ACh_ channels in cardiomyocytes. Arf6—ADP ribosylation factor 6; KCNJ3—mRNA for Kir channels; Kir3.1—channel protein; PIP2—phosphatidylinositol 4,5-bisphosphate; PIP5K—phosphatidylinositol 4-phosphate 5-kinase alpha. Red arrows represent downregulation, while green arrows indicate upregulation.

Morishima et al. identified a significant upregulation of miR-30d in cardiomyocytes from patients with AF, accompanied by a concurrent downregulation of KCNJ3/Kir3.1 at both the mRNA and protein levels (Figure 3A). Transfection experiments using the miR-30 precursor resulted in diminished *I*_KACh_ currents, whereas knockdown of miR-30d led to enhanced expression of KCNJ3/Kir3.1 [62]. These findings suggest that Ca^2+^ overload in cardiomyocytes with AF induces an increase in miR-30d expression through Ca^2+^-dependent conventional PKC, subsequently suppressing the expression of its target gene, KCNJ3 (Figure 3A) [62].

Additionally, Chidipi and co. investigated the potential impact of cigarette smoke on increasing *I*_KACh_ currents via signaling pathways involving phosphatidylinositol 4-phosphate 5-kinase alpha (PIP5K) and ADP ribosylation factor 6 (Arf6), thereby contributing to the perpetuation of AF (Figure 3B) [63]. Previous studies have indicated that Arf6 facilitates the recruitment and activation of PIP5K at the plasma membrane [64,65], leading to increased production of phosphatidylinositol 4,5-bisphosphate (PIP2), a critical component necessary for the activation of K_ACh_ channels (Figure 3B). The findings of Chidipi et al. support this hypothesis, revealing an additional regulatory mechanism of *I*_KACh_ currents that could influence the pathophysiology of AF [63]. These studies collectively underline the intricate network of genetic and environmental factors influencing the modulation of ion channel activities in cardiac cells.

## 3. Vagal and Cholinergic Influences on AF

### 3.1. Multifaceted Role of Vagal Stimulation in AF Pathogenesis and Cardiac Regulation

Vagal nerve stimulation plays a crucial role in the initiation and maintenance of AF, a mechanism well-documented through its significant influence on the atrial refractory periods and the functionality of the SA and AV nodes. This influence results in pronounced, non-uniform alterations that facilitate the initiation and maintenance of re-entry circuits, a key mechanism in AF pathogenesis [66]. Re-entry occurs when an excitation wave navigates around structural or functional barriers, enabled by shortened effective refractory periods or reduced conduction velocity, thus perpetuating AF. Activation of K_ACh_ channels through ACh infusion notably shortens the APD, reducing the refractory period and potentially enabling AF induction [66,67]. Studies involving human subjects and canine right atrium models have demonstrated that the shortening of the effective refractory period correlates with the persistence of AF, highlighting the complex interplay between the vagal tone, cholinergic stimulation, and electrophysiological properties predisposing individuals to AF development [68,69,70].

Moreover, an autonomic imbalance, characterized by reduced vagal activity and increased sympathetic activity, is implicated in various pathological cardiac conditions such as heart failure, arrhythmia, ischemia, and hypertension [71]. The differential innervation of the heart by the right and left branches of the vagus nerve, selectively influencing the atria and ventricles, respectively, underscores the nuanced regulation of cardiac electrophysiology through complex interactions between M_2_R and the adenosine A1 receptor (A_1_R), which converge on the ACh-gated inward rectifier signaling pathway via G_i/o_ proteins. These interactions modulate the heart rate, affect APD, and adjust the refractory period [32,71,72].

Extending beyond primary cardiovascular actions, the regulatory functions of the vagus nerve encompass a spectrum of physiological effects that contribute to cardiac health [71]. These include modulating anti-inflammatory responses, enhancing nitric oxide (NO) production, improving mitochondrial function, and regulating Ca^2+^ currents. Prolonged vagal stimulation, for instance, has been linked to increased expression of endothelial NO synthase (eNOS), facilitated through the phosphatidylinositol 3-kinase/serine-threonine specific protein kinase (PI3K)/Akt signaling pathway, mediated by either M_2_ or M_3_ receptors. This comprehensive view emphasizes the intricate role of the vagus nerve in cardiac function modulation and overall cardiovascular health [71,73].

### 3.2. Vagal Influence on Ca^2+^ Currents and Sinus Node Pacemaking

The modulation of Ca^2+^ currents within the pacemaker cells of the sinus node is intricately regulated by parasympathetic stimulation through M_2_ receptors. This regulation is mediated by two principal pathways: the activation of G-protein-coupled K_ACh_ and the involvement of the alpha-i subunit of G-proteins. Such activation leads to a decreased intracellular concentration of cyclic adenosine monophosphate (cAMP) and protein kinase A (PKA), resulting in diminished Ca^2+^ inflow through Cav1.2 and Cav1.3 and a reduction in the hyperpolarization-activated cyclic nucleotide-gated (HCN) channels, known as the funny channels. This cascade of events effectively inhibits sinus node automatism, subsequently decreasing the heart rate [74].

PKA plays a crucial role in cardiomyocyte function, primarily through the phosphorylation of various proteins. Notably, PKA phosphorylates ion channels are integral to pacemaker depolarization, such as the funny channels. This action enhances Ca^2+^ influx and contributes to AP shortening through one or more delayed rectifier potassium currents (*I*_KDR_), underscoring the significance of PKA in the regulatory mechanisms of cardiac electrophysiology [75].

## 4. Genetic Factors and Insights into AF

### 4.1. Genetic Insights into AF

A comprehensive meta-analysis of genome-wide association studies (GWASs) on AF has identified 97 loci associated with the disorder (Table 1). Transcriptome analysis further elucidated 57 genes linked to AF, with 42 situated within these previously identified loci (Table 1). These genes predominantly influence the heart structure, electrophysiology, and cardiomyocyte functionality [76].

In European populations, the analysis revealed three distinct loci adjacent to or within the genes cyclin-dependent 6 kinase (CDK6), Ephrin type-A receptor 3 (EPHA3), and Golgi-soluble NSF attachment protein receptor complex member 2 (GOSR2). The most robust association across European, African, American, and Japanese cohorts was located on chromosome 4q25, proximal to the paired-like homeodomain transcription factor 2, also known as the pituitary homeobox 2 (PITX2) gene. This region also harbors a missense variant in coiled-coil domain-containing protein 92 (CCDC92), also known as Limkain beta-2, which is implicated in cardiac tissue damage and associated with coronary artery diseases, as well as a reduction in paired related homeobox 1 (PRRX1) expression that contributes to AF. Notably, the majority of detected variants are situated in non-coding regions, with 64 loci identified within regulatory elements, highlighting the critical role of non-coding DNA in AF pathology (Table 1) [77]. Mutations in genes responsible for the production of the connexins have also been linked to the development of AF. Mutations in GJA1, which encodes connexin 43, have been linked to conduction defects and an increased risk of AF [78]. Similarly, mutations in GJA5, which encodes connexin 40, have been associated with familial types of AF, emphasizing their critical role in maintaining proper atrial conduction [79].

Several genes associated with AF exhibit pleiotropic effects. For instance, castor zinc finger 1 (CASZ1) not only correlates with AF but also with the incidence of hypertension. Expanded analyses have further extended the list of AF-associated loci to 134, enriching our understanding of AF’s etiology. This includes loci targeted by antiarrhythmic medications, such as sodium voltage-gated channel alpha subunit 5 (SCN5A) and potassium voltage-gated channel subfamily H member 2 (KCNH2), and emphasizes the significance of transcriptional regulation in AF, with genes like T-box transcription factors 3 and 5 (TBX3 and TBX5), NK2 homeobox 2 (NKX2) and PITX2 being implicated (Table 1) [76].

Many identified loci are also associated with Mendelian forms of arrhythmia, suggesting a genetic predisposition to various hereditary cardiomyopathies and cardiac conduction system disorders. AF is prevalent among patients with inherited conditions, such as those with mutations in the protein kinase AMP-activated non-catalytic subunit gamma 2 (PRKAG2) gene linked to hypertrophic cardiomyopathy [80]. A mutation in the potassium voltage-gated channel subfamily Q member 1 (KCNQ1) gene on chromosome 11p15.5, associated with AF, affects the shortening of the APD and effective refractory periods, thus facilitating AF maintenance. Loci near zinc finger homeobox 3 (ZFHX3) on chromosome 16q22 have also been implicated in AF [80,81] (Table 1).

Furthermore, a missense variant (c.G681C, p.E227D, rs1477078144) in the dystrobrevin alpha (DTNA) gene associated with early-onset AF affects the Rho-(RAS homolog) signaling pathway. Variants in DTNA and the sodium voltage-gated channel alpha subunit 1 (SCN1A) gene underscore the genetic complexity underlying AF [82].

### 4.2. Overview of Potassium Inwardly Rectifying Channel Subfamily J Members 3 and 5 (KCNJ3 and KCNJ5) Mutations and Their Impact on AF Development

Recent investigations have elucidated the significant role of mutations in the subunit genes of the K_ACh_ channel, specifically potassium inwardly rectifying channel subfamily J members 3 and 5 (KCNJ3 and KCNJ5), in the onset and progression of AF in humans. A noteworthy familial case study identified a causative mutation in KCNJ3 (c.247A>C), which is associated with sinus node dysfunction and AF. Additionally, a comprehensive genetic analysis involving 2185 unrelated individuals revealed 5 heterozygous mutations across KCNJ3 and KCNJ5. These mutations are KCNJ3 c.253T>C (p.F85L), KCNJ3 c.1486A>C (p.N496H), KCNJ5 NM_000890: c.785A>G (p.D262G), KCNJ5 c.907G>A (p.V303I), and KCNJ5 c.1159G>A (p.G387R). Notably, mutations KCNJ5 p.D262G and KCNJ5 p.V303I are predicted to be pathogenic [83].

The mutation KCNJ3 p.N496H is associated with an elevated risk of AF development, while mutations KCNJ5 p.G387R and KCNJ3 p.F85L are considered either neutral or potentially beneficial with respect to disease progression. In cases where the Kir3.1 mutation (KCNJ3 c.247A>C, p.N83H) is present, basal channel activity without ACh stimulation was observed to be double that of subjects possessing the wild-type Kir3.1, indicating an overall enhancement in channel function [83]. This refined genetic insight underscores the complex relationship between specific mutations and their impact on the physiological mechanisms underlying AF development.

## 5. Impact of Physical Activity on AF Vulnerability and the Role of RGS4

Recent studies have demonstrated that increased physical activity is associated with an elevated vulnerability to AF, primarily due to an enhancement of sinus rhythm facilitated by an increased vagal tone. This augmented sensitivity of the K_ACh_ channels to cholinergic stimulation following exercise is mediated by the downregulation of signaling pathways involving proteins from the RGS superfamily, particularly RGS4. This protein is predominantly synthesized in the myocytes of the sinoatrial node [50]. RGS4 serves as a critical modulator of K_ACh_ channels, which are essential for the regulation of the heart rate. Empirical research involving RGS4-deficient mice has indicated a pronounced increase in the response to M_2_ receptor activation, which is apparent not only in the intact animal but also in the isolated heart and sinoatrial node myocytes. These findings suggest that RGS4 plays a pivotal role in the modulation of sinus rhythm by attenuating parasympathetic signaling and the activity of K_ACh_, thereby influencing the susceptibility to AF [48,84].

## 6. Therapeutic Perspectives on Muscarinic Receptor Associated Currents

From a therapeutic perspective, the selective inhibition of currents mediated by muscarinic receptors offers a promising approach for treatment, particularly due to the predominance of ACh-controlled *I*_KACh_ currents in the atria compared to the ventricles. The application of selective blockers has revealed that not only K_ACh_ but also Kir channels are inhibited in cardiomyocytes. These findings hold significant clinical importance because of the potential proarrhythmic effects.

Further experimentation with various selective blockers has indicated a distinct mechanism of K_ACh_ inhibition that operates exclusively through extracellular receptors. On the contrary, the intracellular administration of these blockers does not affect K^+^ conductivity within the cell [85]. This specificity underscores the therapeutic potential of targeting extracellular receptor pathways in the modulation of cardiac electrophysiology and arrhythmia management.

## 7. Future Directions and Conclusion

The research delineated in this manuscript opens several avenues for further exploration of the role of K_ACh_ channels in AF. Future studies should focus on the detailed molecular interactions between K_ACh_ channels and their regulatory proteins, especially under varying physiological and pathophysiological conditions. Additionally, the genetic basis of K_ACh_ channel functionality and its implications for AF susceptibility present a promising field of inquiry. Investigating the genetic predispositions that affect channel behavior could lead to personalized medicine approaches that adapt AF therapy to individual genetic makeups.

Moreover, the development of selective K_ACh_ channel blockers holds substantial potential for clinical application. These blockers should be designed to target specific channel activities without disrupting other cardiac functions. Clinical trials to evaluate the efficacy and safety of these targeted therapies will be crucial. Such studies could significantly advance our understanding of the therapeutic potential of K_ACh_ modulation in AF management.

In conclusion, this review has highlighted the critical role of K_ACh_ channels in the initiation and maintenance of AF, underscoring their potential as therapeutic targets. As the research progresses, a deeper understanding of the complex interactions governing K_ACh_ channel activity and its genetic regulation could ultimately lead to more effective and precise interventions for AF, improving outcomes for patients with this challenging cardiac condition.

## Figures and Tables

**Table 1:** Genetic mutations associated with AF development.

Category	Details
GWAS Findings	97 loci associated with AF
Transcriptome Analysis	57 genes linked to AF; 42 genes within identified loci
European Populations	Loci near CDK6, EPHA3, GOSR2
Strongest Association	Chromosome 4q25 (near PITX2); CCDC92 variant; reduced PRRX1 expression
Non-Coding Regions	64 loci in regulatory elements
Pleiotropic Effects	CASZ1 gene (associated with AF and hypertension)
Expanded Loci List	134 loci associated with AF; includes SCN5A, KCNH2, TBX3, TBX5, NKX2 genes
Connexines Association	GJA1, which encodes connexin 43 and GJA5, which encodes connexin 40
Mendelian Forms and Genetic Predisposition	PRKAG2 gene (linked to hypertrophic cardiomyopathy); KCNQ1 gene (affects APD and refractory periods); ZFHX3 gene
Specific Genetic Vari-ants	DTNA variant (affects Rho signaling pathway); SCN1A gene variant

Abbreviations: CASZ1—castor zinc finger 1; CCDC92—coiled-coil domain-containing protein 92; CDK6—cyclin-dependent 6 kinase; DTNA—dystrobrevin alpha gene; EPHA3—EPH receptor A3; GJA1 and GJA5—genes encoding connexin 43 and 40; GOSR2—Golgi SNAP receptor complex member 2; KCNH2—voltage-gated channel subfamily H member 2; KCNQ1—potassium voltage-gated channel subfamily Q member 1; NKX2—NK2 homeobox 2; PITX2—pituitary homeobox 2; PRKAG2—protein kinase AMP-activated non-catalytic subunit gamma 2; PRRX1—paired related homeobox 1; SCN1A—sodium voltage-gated channel alpha subunit 1; SCN5A—sodium voltage-gated channel alpha subunit 5; TBX3 and TBX5—T-box transcription factors 3 and 5; ZFHX3—zinc finger homeobox 3.

## Data Availability

Not applicable.
